# Punicic Acid: A Potential Nutraceutical Compound in Pomegranate Seed Oil and Its Cardiovascular Benefits

**DOI:** 10.3390/foods14142412

**Published:** 2025-07-08

**Authors:** Manal Almoraie, Jeremy Spencer, Carol Wagstaff

**Affiliations:** 1 Department of Food and Nutritional Sciences, University of Reading, Whiteknights, Reading RG6 6DZ, UK; c.wagstaff@reading.ac.uk; 2 Department of Food Science and Nutrition, Faculty of Human Sciences and Design, King Abdulaziz University, Jeddah 21589, Saudi Arabia

**Keywords:** pomegranate seed oil, punicic acid, pharmacological, consumption, nutraceutical

## Abstract

Pomegranate seed oil (PSO) is rich in punicic acid (PA), a conjugated isomer of α-linolenic acid, and exhibits a range of pharmacological properties. Given the significant role of nutraceuticals in the prevention of various diseases, PA stands out as an important phytoconstituent within this category. This review aimed to examine the composition of PSOs and their positive effects on cardiovascular risk factors. PA possesses potent antioxidant and anti-inflammatory effects, as well as aids in managing obesity and diabetes while improving lipid profiles. Additionally, the diverse cardiovascular health benefits associated with PSO consumption are detailed. There are various health benefits that are derived from PSO consumption; however, despite these promising findings, there remains a critical need for further clinical studies to validate these effects.

## 1. Introduction

Cardiovascular disease (CVD) is the main cause of death globally; in the UK, it accounts for around 25% of overall deaths [1,2]. The incidence and severity of CVD are significantly influenced by fat and unhealthy eating habits. Saturated fats raise serum total cholesterol (TC) and low-density lipoprotein (LDL) cholesterol, increasing the risk of CVD. Around 31% of CVD and 11% of strokes worldwide are linked to diets high in saturated fat [3]. Additionally, the source and quality of dietary fat have been confirmed as being more significant for preventing CVD compared to the overall quantity of fat [4]. Current recognised dietary recommendations to reduce the risk of CVD include avoiding trans-fatty acids (TFAs), eating fish and plant sources of polyunsaturated fatty acids (PUFAs), and reducing saturated fat intake [5,6].

Pomegranate (*Punica granatum* L., Lythraceae) is a nutrient-rich fruit widely valued for its edible arils and therapeutic properties. Beyond its juice, recent research has highlighted the potential of its by-products, particularly the rind and seeds, as sources of bioactive compounds with applications in food, medicine, and cosmetics [7]. The seeds of the pomegranate (PS) comprise around 20% of the fruit’s weight and are the main by-product of pomegranate juice extraction [8,9]. The estimated amount of annual PS waste is around 1.62 million tons. PSs have tremendous nutritional value [10], and the extraction of the compounds they contain increases the pomegranate’s economic and health benefits while also reducing waste [11].

Numerous pharmacological advantages of PSs have been demonstrated, including anticancer and antiosteoporotic properties, along with other benefits from its components, including proteins, sterols, unsaturated fatty acids, tocopherols, and phenols [12,13]. Pomegranate seed oil (PSO), which accounts for 12–20% of the total PS weight, is high in punicic acid (PA), a rare conjugated linolenic acid (CLnA) isomer with a variety of pharmacological properties [14,15]. Its primary characteristics include anti-inflammatory activity, protection of liver and kidney function, anticancer effects, immune system enhancement, glucose metabolism improvement, insulin resistance reduction, and improved lipid profiles [16,17,18]. Recent studies underscore the potential of PSO not only as a functional therapeutic agent [19] but also as a sustainable product that transforms agricultural waste into valuable health-promoting resources [11]. However, the complete value of PSs remains underutilised, owing to the limited understanding and insufficient development of their applications. While several studies have examined the chemical composition of PSs [20,21], comprehensive and systematic analyses of the pharmacological effects of PSO are still lacking. This highlights the need for in-depth reviews that consolidate existing knowledge and encourage further exploration into the pharmaceutical, nutraceutical, food, and allied sectors. This review examines the beneficial effects of PA as a novel nutraceutical component that has recently gained growing attention for its potential to reduce CVD risk factors.

## 2. Methodology of Literature Review

A structured literature search was performed to provide a comprehensive and updated overview of the cardiovascular effects and therapeutic potential of PA. The PubMed, Scopus, Web of Science, and Google Scholar databases were searched for relevant publications between November 2004 and July 2024. The key words included various combinations of the following: ‘punicic acid’, ‘pomegranate seed oil’, ‘conjugated linolenic acid’, ‘cardiovascular’, ‘atherosclerosis’, ‘endothelial function’, ‘oxidative stress’, ‘inflammation’, ‘hypertension’, ‘lipid profile’, ‘metabolism’, ‘bioactive lipids’, and ‘health benefits’.

Only peer-reviewed articles published in English were considered. The inclusion criteria focused on experimental and clinical studies evaluating PA’s biological activities, especially those linked to cardiovascular health, such as anti-inflammatory, antioxidant, lipid-lowering, and vascular effects. Both in vitro and in vivo models, as well as human trials and systematic reviews, were included. This review is structured to cover several core aspects: (1) the chemical structure, biosynthesis, and natural sources of PA; (2) the metabolic fate of PA and its biological transformation pathways in mammals; (3) the cardiovascular benefits of PA, analysed through mechanistic and experimental evidence, including effects on the lipid profile, antidiabetic activity, inflammation, and antioxidant protection; and (4) a comparative analysis of PA with other well-known bioactive fatty acids, notably omega-3 PUFAs, in the context of cardiovascular protection.

## 3. Structure, Biosynthesis, and Natural Sources of PA

Methylene-interrupted double bonds in carbon chains define the structure of most PUFAs. A conjugated structure is formed, and the resulting fatty acid is known as a conjugated fatty acid (CFA) if the methylene group between these two bonds is eliminated. CFAs are geometric and positional isomers of PUFAs that can include dienes, trienes, and tetraenes [22]; this unique structure impacts their specific chemical properties and physiological activity [23]. In nature, CFAs typically contain at least one trans-double bond and are classified as TFAs, which are divided into two groups: conjugated linoleic acids (CLAs) and CLnAs. CLAs are prevalent in products obtained from ruminants, such as meat, milk, and dairy, while CLnAs are abundant in many dietary oils made from plants [24,25].

**Figure 1:**
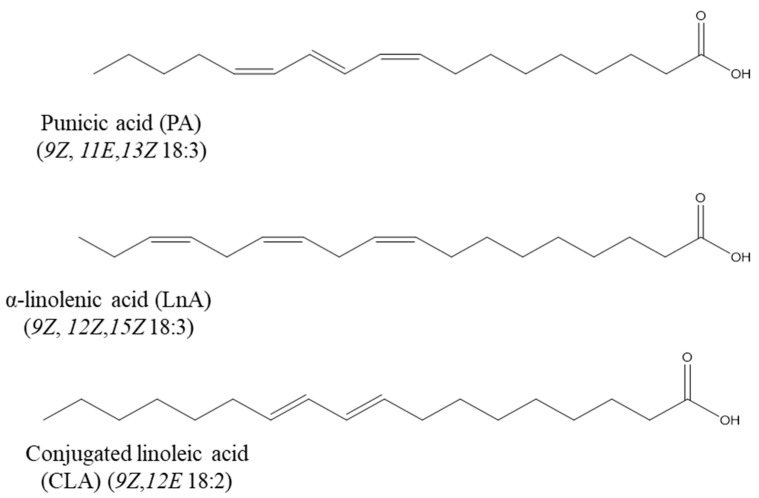
Comparisons between the chemical structures of punicic acid, α-linolenic acid, and conjugated linoleic acid, adapted from [29]. Chemical structures drawn in ChemDraw (version 21).

PA, one of the most well-known CLnA isomers, is a conjugated triene with double bonds at positions (9Z, 11E, 13Z, 18:3); it is the most abundant fatty acid present in PSO [26] (approximately 64–83% of total fatty acids) [17,27]. Theoretically, PA features 66% *Z*-type double bonds and 33% *E*-type double bonds [28]. Figure 1 shows that PA is an isomer of CLnA and has a similar structure to several other fatty acids, such as CLA and α-linolenic acid (α-LnA) [29].

The primary source of CLnA is plant synthesis, which mostly takes place during the desaturation and conjugation of fatty acids at the sn-2 position of phosphatidylcholine. Fatty acid desaturases 2 and 3 catalyse the reaction of oleic acid at the sn-2 position in phosphatidylcholine in plants that produce conjugated fatty acids (such as *P. granatum* (pomegranate), *Momordica charantia* L., *Cucurbitaceae* (bitter gourd), and *Calendula officinalis* (calendula)), sequentially forming linoleic acid (LA) and α-LnA. These intermediates are then converted by fatty acid conjugases into CLnA isomers [30,31]. CLnA can also be found in animal-derived products, though in lower amounts [32].

The major sources of PA include *P. granatum*, *Fevillea trilobata* L., *Cucurbitaceae* [33,34], *Momordica balsamina* L., *Cucurbitaceae* [35], *Trichosanthes anguina* L., *Cucurbitaceae* [36], and *Trichosanthes kirilowii* Maxim., *Cucurbitaceae* [37]. Jacaric acid is found in the sed oil of *Jacaranda mimosifolia* D. Don, *Bignoniaceae* [38], while α- and β-calendic acids are primarily obtained from *Calendula officinalis* L., *Asteraceae* [39]. Catalpic acid is found in *Catalpa ovata* G. Don, *Bignoniaceae* [40], and *Catalpa bignonioides* Walt., *Bignoniaceae* [41]. Some CLnA isomers are also present in dairy products. For instance, α-rumelenic acid occurs in bovine milk [42], beef [43], and goat meat [44]. Additionally, ruminants can produce isomers, such as C18:3 Δ9 Z,11 E,15 Z and C18:3 Δ9 Z,13 E,15 Z, through microbial biohydrogenation. Notably, plant-derived CLnA isomers are typically conjugated trienes, whereas ruminant-derived isomers are mostly conjugated dienes. Table 1 summarises the main natural sources and contents of CLnA found in plant seeds.

## 4. Metabolism of Punicic Acid

**Figure 2:**
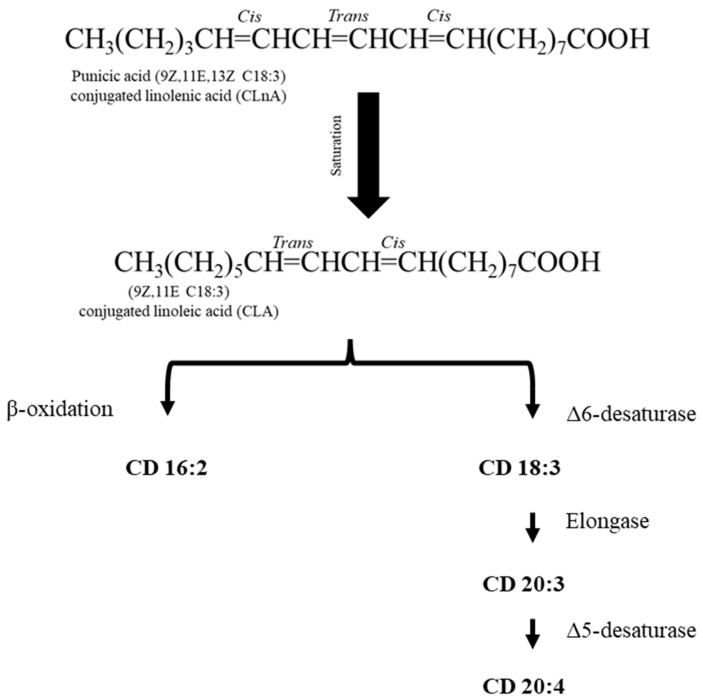
Punicic acid metabolism, adapted from [53]. Punicic acid is first converted into conjugated linoleic acid (CLA, specifically the 9Z, 11E isomer), which is then either broken down via β-oxidation into conjugated diene 16:2 (CD 16:2) or undergoes further metabolism by the enzyme Δ6-desaturase to form CD 18:3. This intermediate can then be elongated and desaturated to produce CD 20:3 and CD 20:4. Chemical structures drawn in ChemDraw (version 21).

There is limited data available regarding the metabolism of PA. Findings from trials indicate that it is primarily metabolised in the liver, where it proceeds through a saturation process to form the CLA isomer 9Z, 11E. This CLA isomer can follow two major metabolic paths while maintaining its conjugated diene structure (Figure 2). The first pathway involves β-oxidation, leading to the formation of conjugated diene (CD) 16:2. Alternatively, it is desaturated by the enzyme Δ6-desaturase to produce CD 18:3. This intermediate is then elongated to CD 20:3 and further desaturated by Δ5-desaturase to yield CD 20:4 [46]. Notably, PA is absorbed in the intestines of rodents and can be transformed into 9Z, 11E, 18:2 in several rat tissues, including the brain, liver, adipose tissue, and plasma [47,48]. In a human trial, Yuan et al. [49] demonstrated that PA may be integrated into erythrocytes and plasma and that a portion of it can be converted into 9Z and 11E, 18:2. The consumption of *T. kirilowii* seed kernels containing 3 g PA/day for 28 days resulted in the amount of PA (c9t11c13) in plasma and red blood cell membranes increasing to 0.47% and 0.37%, respectively; moreover, the levels of 9Z, 11E, 18:2 in plasma and red blood cell membranes increased from 0.05% to 0.23% and 0.03% to 0.17%, respectively.

Schneider et al. [50] investigated the absorption and metabolism of CLnA into CLA using Caco-2 cells as an intestinal barrier model. The findings demonstrated that the shape of the Δ13 double bond affected the efficiency with which Caco-2 cells absorb and transform CLnA into CLA. Additionally, the quantity of trans-double bonds was linked to the distribution of CLnA between neutral lipids and phospholipids; higher trans-bonds led to a higher accumulation in the neutral lipid fraction. Another theory explains the metabolic conversion of CLnA to CLA by nicotinamide adenine dinucleotide phosphate, which is an enzyme for conjugated trienoic acid reorganisation, in which it catalyses the Δ13 double bond saturation events that cause the metabolic conversion of CLnA to CLA [50]. In another study, mice administered 1% PA for six weeks had larger levels of omega-3 in their liver phospholipids than mice given 1% alpha-eleostearic acid [51]. Similar outcomes were obtained in another study where 0.5% PA from PSO was added to the diet [52]. There is a critical need for well-designed human studies to elucidate the metabolic fate and bioavailability of PA in clinical settings.

## 5. Cardiovascular Benefits of PA

### 5.1. Cardiovascular Protection

CVD is defined by both macrovascular and microvascular alterations, including conditions such as atherosclerosis, hypertension, and endothelial dysfunction [54]. Several studies have investigated the cardioprotective properties of PSO and have suggested its beneficial role in vascular function and blood pressure regulation. For example, vasodilation effects were observed in an ex vivo study using rat thoracic aorta rings, where PSO induced endothelium-dependent relaxation via the nitric oxide–guanylyl cyclase signalling pathway, independent of other inhibitory pathways. This was accompanied by a slight reduction in both systolic and diastolic blood pressure, along with a mild decrease in heart rate following administration [55].

Yılmaz et al. [56] clarified the mechanism of PSO-induced vasodilation in isolated rat thoracic aorta rings. The study revealed that PSO induced a concentration-dependent relaxation in endothelium-intact rings but had no effect in those lacking an endothelium. The vasorelaxant response was significantly inhibited by L-NAME and ODQ, confirming the involvement of the nitric oxide, guanylyl cyclase pathway. Interestingly, this effect was not altered by potassium channel blockers (TEA, 4-AP, glibenclamide) nor by agents affecting prostanoids (indomethacin), β-adrenergic receptors (propranolol), or the renin-angiotensin system (losartan and captopril). A slight decrease in systolic and diastolic blood pressure, along with a reduced heart rate, was also observed in vivo. These findings reinforce the central role of endothelial nitric oxide signalling in PSO-induced vasodilation and help rule out alternative pathways.

Radjabian et al. [57] reported that the progression of atherosclerosis in hypercholesterolemic rabbits was significantly reduced in a group treated with PSO compared to controls. However, conflicting results were noted in the study by Franczyk-Żarów et al. [19], which found no significant anti-atherosclerotic effects. These discrepancies may stem from variations in the experimental design, including differences in animal species, diet composition, PSO dose, and treatment duration. Such inconsistencies underscore the need for standardised protocols for the better assessment of the cardiovascular effects of PSO. Bihamta et al. [58] investigated the protective effects of PSO against oxidative stress in H9c2 cardiomyocytes exposed to hydrogen peroxide (H_2_O_2_). Pretreatment with PSO (up to 200 μg/mL) significantly improved cell viability and reduced intracellular reactive oxygen species (ROS) levels compared to H_2_O_2_-treated cells. Additionally, PSO enhanced cellular antioxidant defence, as demonstrated by a significant increase in superoxide dismutase (SOD) activity at concentrations of 50, 100, and 200 μg/mL. These findings suggest that PSO exerts antioxidant effects by both reducing ROS accumulation and upregulating endogenous antioxidant enzymes.

In human trials, dietary supplementation with PA, a major constituent of PSO, significantly reduced systolic (*p* < 0.01) and diastolic (*p* < 0.05) blood pressure in overweight female participants over a 13-week intervention period [59]. However, this effect was small and gender-specific, limiting its generalisability across populations.

### 5.2. Lipid Reduction Effects

High blood lipid levels, commonly referred to as hyperlipidaemia, can elevate the risk of CVD [60]. PSO was demonstrated to reduce triacylglycerol (TAG) levels in the plasma lipids of hypercholesterolemic rats [61]. In that study, PSO intake improved plasma lipid profile, reducing TC, triglyceride (TG), and LDL-C levels compared to a control. Mukherjee et al. [36] investigated the effect of various concentrations of PA (0.6%, 2.1%, and 2.4%) and found that a group treated with 2.4% PA had significantly lower LDL-C and plasma cholesterol levels. In contrast, Yang et al. [62] indicated that PSO does not affect the level of serum cholesterol. In another study, Yamasaki et al. [63] found that meals containing 0.12% or 1.2% PSO, when eaten for three weeks, significantly raised serum levels of phospholipids and TAG, but not TC, in PSO-treated groups.

Yuan et al. [47] investigated the impact of PA and α-eleostearic acid (9Z, 11E, 13E CLnA; α-ESA) on body fat in ICR mice; a 6-week feeding trial with a diet supplemented with 1% α-ESA and PA resulted in a significant reduction in hepatic TAG content. Additionally, a study on Otsuka Long Evans Tokushima Fatty (OLETF) rats revealed that PA inhibited delta-9 desaturation, reducing hepatic TAG accumulation [64]. Similarly, a study by Teh et al. [65], comparing solvent-extracted and expeller-pressed pomegranate, tomato, and grape seed oils in hamsters, demonstrated that PSO significantly reduced plasma TG, VLDL, and LDL-C levels, along with improvements in LDL/HDL ratios. However, the hepatic lipid content remained unchanged, suggesting that PSO effects may be more substantial in circulating lipid modulation rather than liver fat metabolism. These findings support the lipid-lowering potential of PSO, though differences in the outcomes across studies underscore the need for further research with standardised dosages and experimental conditions.

PSO also effectively decreased lipid accumulation in HepG2 cells and 3T3-L1 differentiated adipocytes [66]. In broilers, PSO significantly reduced TC levels [67]. Moreover, PA markedly suppressed apolipoprotein (Apo)B100 secretion in human HepG2 cells in vitro [68]. ApoB100, a crucial component of very low-density lipoprotein, was positively associated with the risk of coronary heart disease and atherosclerosis [68].

Further, supplementation with PSO has positive outcomes in reducing diet-induced obesity in rats. Vroegrijk et al. [27] tested the effects of PSO on high-fat diet-induced obesity in mice; PSO supplementation over 12 weeks reduced body weight (30.5 vs. 33.8 g, *p* = 0.02) and body fat mass (3.3 vs. 6.7 g, *p* = 0.02) and was the main cause of weight loss. Additionally, PSO controls body weight increases in mice fed a high-fat diet by upregulating the expression of the gene uncoupling protein 1, which is linked to brown adipose tissue. Moreover, it induces the formation of beige-like tissue in white adipose tissue, thereby influencing abdominal fat mass and ratio [69]. Arao et al. [64] also examined the effects of PSO in obese hyperlipidaemic OLETF rats; after treatment with 5% PSO, the weight of omental white adipose tissue was significantly reduced. The decrease in total weight gain was found to be due to a lower concentration of leptin and a rise in the plasma concentration of adiponectin with a regimen of PSO intake [70].

Studies examining human subjects are scarce (Table 2). However, the consumption of PSO in hyperlipidaemic subjects was evaluated by Mirmiran et al. [71] in a double-blind, placebo-controlled randomised clinical trial, where the treatment group received 400 mg of PSO twice a day for 4 weeks. PSO consumption had encouraging effects on lipid profiles, such as TG and the TG–high-density lipoprotein (HDL)-C ratio. Similarly, Asghari et al. [72] found that PSO consumption reduced TG. However, an interventional study, in which 3 g of PA was administered to healthy young subjects for 28 days, showed no effect on weight reduction or the serum lipid profile; while urinary 8-iso-prostaglandin F2α levels increased significantly, there was no notable impact on cholesterol-reactive proteins, insulin, glucose, or insulin resistance [49]. Overall, the beneficial effects of PA are particularly evident in its role in regulating lipid metabolism homeostasis during fat reduction processes. Although PA demonstrates improvement in lipid metabolism in animal models, human evidence remains limited and inconsistent. These inconsistencies may stem from variations in the study design, dosing, and baseline metabolic status of subjects. Moreover, the exact molecular mechanisms by which PA influences lipid metabolism, such as its effect on ApoB100 and adipokine signalling, require further clarification. As such, although PA demonstrates potential in reducing lipid levels, its clinical significance and translational value are yet to be firmly established. More well-controlled clinical trials are warranted to confirm its efficacy and clarify the underlying mechanisms.

### 5.3. Antidiabetic Effects

Diabetes is a risk factor for CVD [73]. Elevated blood sugar levels over time in diabetes can harm arteries, increasing the likelihood that fatty deposits will form. An almost two-fold increase in the risk of CVD is linked to diabetes and high blood glucose [74]. Notably, PSO had potentially positive effects on insulin intolerance and diabetes in several in vivo and in vitro models [75,76]. PS extract reduced fasting blood glucose levels in rats with streptozotocin-induced type 2 diabetes, which, in turn, decreased the prevalence of insulin resistance and obesity [77]. However, Nekooian et al. [78] found that feeding PSO to the same model at 200 and 600 mg/kg/day for 28 days raised serum insulin levels but showed no difference in blood glucose levels. The reason for the rise in blood insulin is likely that PA regulates peroxisome proliferator-activated receptor gamma (PPAR-γ)-sensitive genes [79]. Bassaganya-Riera et al. [80] found that PA can be used as a safer alternative to synthetic drugs in the prevention and treatment of metabolic and inflammatory diseases.

Further, in a study by Harzallah et al. [81], who treated diabetic mice with 2 mL/kg/day PSO for 6 weeks, positive effects were detected. Specifically, the findings demonstrated a considerable improvement in insulin sensitivity indicators and a significant drop in fasting blood glucose. Additionally, PSO improved insulin sensitivity in rats fed a high-fat diet [27]. A similar trial by Miranda et al. [82] fed rats a diet including 0.5% PA; they found no change in insulin resistance, but glycaemic values significantly decreased in the PA group. McFarlin et al. [70] found that PSO intake was linked to an improvement in insulin sensitivity, which may have decreased the risk of type 2 diabetes.

In vitro studies have indicated additional mechanisms underlying PA activity. Anusree et al. [83] investigated the impact of tumour necrosis factor (TNF)-α on 3T3-L1 adipocyte insulin resistance and mitochondrial dysfunction. PA administration (5, 10, and 30 μM) increased mitochondrial biogenesis and energetics, decreased ROS generation, and improved glucose absorption in insulin-resistant cells; it also prevented changes in mitochondrial proteins linked to 3T3-L1 adipocyte dysfunction. Additionally, incubation with PA promoted adiponectin secretion and increased both the expression and translocation of glucose transporter type 4 (GLUT4) in adipocytes, possibly acting as a PPARγ agonist [84]. Importantly, PPAR-γ agonists promote mitochondrial biogenesis and restore the mitochondrial fission–fusion ratio, which is impacted by inflammation and elevated TNF-α [85].

In a clinical study (Table 2), Seyed Hashemi et al. [86] administered 10 g of PS powder for 8 weeks in patients with type 2 diabetes mellitus, resulting in significantly decreased fasting blood glucose and glycated haemoglobin levels compared to a placebo. Additionally, Khajebishak et al. [87] examined the effects of administering 3 g PSO daily to patients with obesity and type 2 diabetes. After 8 weeks, PSO resulted in notable reductions in serum levels of fasting blood glucose. However, Faghihimani et al. [88] found no change in fasting blood sugar, insulin resistance, or the lipid profile. Overall, these findings suggest that PA is a promising nutraceutical for managing metabolic syndrome and diabetes. Although PA has demonstrated potential antidiabetic effects in both animal models and in vitro studies, primarily by improving insulin sensitivity, enhancing glucose uptake, and modulating mitochondrial function, clinical outcomes remain inconsistent. While some human trials report improvements in the fasting blood glucose levels, others demonstrated no significant metabolic benefits. These discrepancies may be attributed to variations in the dosage, duration, patient populations, and PA formulations. More rigorous, long-term clinical trials are warranted to determine its true efficacy and translational relevance in diabetes management.

### 5.4. Anti-Inflammatory Effects

Inflammation serves as a central pathogenic factor in the development of CVDs. Targeting specific inflammatory pathways presents a promising therapeutic strategy for managing a wide range of cardiovascular conditions [89]. Changes in serum TGs, HDL-C levels, and TNF-α are closely interrelated. TNF-α plays a role in promoting lipolysis, activating endothelial cells, and inducing vascular damage; it is also strongly associated with the development of insulin resistance [90,91].

Notably, PSO can effectively inhibit the activity or production of inflammatory markers, such as nuclear factor (NF)-κB, TNF-α, and interleukin (IL)-6 [81,92]. Several in vivo and in vitro studies have explored the effects of PA on inflammatory cytokines. A recent study demonstrated that CLnAs have anti-inflammatory properties in a colitis model, significantly increasing levels of PPAR-γ and IL-10, two anti-inflammatory cytokines, while decreasing pro-inflammatory cytokines (TNF-α, IL-1β, and IL-6) [93,94]. PSO was also evaluated for its anti-inflammatory properties in a gastrointestinal in vitro digestion model, where it reduced IL-6, IL-8, and TNF-α production in lipopolysaccharide-stimulated Caco-2 cells [95]. Moreover, a rat model suggested that PA exhibits a strong anti-inflammatory effect by preventing the increase in nicotinamide adenine dinucleotide phosphate oxidase caused by TNF-α [96]. Similarly, this model demonstrated the ability of PSO to prevent necrotising enterocolitis. PSO’s anti-inflammatory effects and restoration of epithelial homeostasis were again found to result from a reduction in TNF-α, IL-6, and IL-8 levels [97]. However, McFarlin et al. [70] found that administering PSO to mice did not significantly change indicators of systemic inflammation, though this could be due to inadequate dosing.

The administration of PA has also been shown to alleviate the effects of diabetes in mouse models through its anti-inflammatory properties by reducing oxidative stress [28]. Taheri Rouhi et al. [98] also observed a significant reduction (*p* ≤ 0.05) in the levels of plasma inflammatory biomarkers (IL-6, NF-κB, and TNF-α), which were actively elevated in diabetic rats before treatment, following the administration of 5 mg/kg body weight of PS powder for 21 days. Additionally, in mice, Hontecillas et al. [79] found that PSO efficiently suppressed obesity-related inflammation through TNF-α suppression, and Yamasaki et al. [63] demonstrated improvement in B-cell function after PSO consumption. Evidence from recent studies indicates that fatty acids, such as PA, suppress the expression of inflammatory genes by inhibiting pathways, such as NF-κB, and activating PPAR α and γ. These actions help regulate immune and inflammatory responses by altering the gene expression involved in cytokine production and immune cell function [99].

PSO may also have potential anti-inflammatory effects in patients with diabetes (Table 2). In a study involving patients with obesity and type 2 diabetes, the administration of PSO for 8 weeks resulted in significant reductions in serum levels of IL-6 and TNF-α. Nonetheless, there were no significant changes in the expression of PPAR-γ [100]. While preclinical studies consistently suggest that PA has anti-inflammatory potential, the evidence in humans remains limited and inconsistent. It is also unclear whether the effects are attributed solely to PA or synergistic components in PSO. These gaps highlight the need for well-designed clinical trials to clarify PA’s mechanisms and efficacy in inflammation-related conditions.

**Table 2:** Human studies investigating the association between pomegranate seed oil and markers of cardiovascular disease risk.

Reference	Source	N (M/F), Age (y)	Subject Group	Study Design	Duration	Treatment	Comparison	Treatment Effect
[49]	Trichosanthes kirilowii (TK) seed (3 g PA)	30 (24 M, 6 F), aged 21–35	Healthy young humans	Randomised controlled trial	28 days	3 g/day	Sunflower seed	↔ lipid profile
[71]	PSO	51 (both), > 20	Hyperlipidaemic subjects (BMI ≥ 35 kg/m^2^, TC < 5.2 mmol/L, TAG > 1.65 mmol/L)	Parallel, randomised, double-blind, and placebo-controlled	4 weeks	400 mg × 2	Placebo	↓ TG: HDL-C ratio ↓ TG ↔ cholesterol and LDL-C
[72]	PSO	51 (both), >20	Hyperlipidaemic	Randomised, double-blind, placebo-controlled	4 weeks	400 mg × 2	Placebo	↔ TNF-α levels↓TG
[88]	PSO	80 (28 M/52 F), 52 ± 6.8	Type 2 diabetes; BMI 20–30	Randomised, double-blind, placebo-controlled	8 weeks	1000 mg × 2	Placebo	↔ FBS, insulin resistance, and lipid profile
[59]	PSO	46 (F)	Patients with breast cancer + overweight; BMI < 35 kg/m^2^	Randomised, double-blind, placebo-controlled	13 weeks	1 g/day	Placebo (350 mg sunflower oil, 350 mg palm olein, and 300 mg corn oil)	↓ SBP and DBP
[87]	PSO	52 (both), 30–50	Patients with obesity and type 2 diabetes	Randomised, double-blind, placebo-controlled	8 weeks	1 g × 3/day	Placebo (paraffin)	↑ Gene expression of GLUT-4 ↓ FBS
[100]	PSO	52 (both), 30–50	Patients with obesity and type 2 diabetes	Randomised, double-blind, placebo-controlled	8 weeks	1 g × 3/day	Placebo (paraffin)	↓ FBS ↓ IL-6 and TNF-α ↔ lipid profile levels
[86]	PSP (as a tea bag in hot water for [10 min])	60 (both), 30–60	Type 2 diabetes BMI > 35	Prospective, double-blind, randomised, placebo-controlled clinical trial	8 weeks	5 g × 2/day	Placebo (*n* = 30) HMWPG	↓ HbA1c ↓ FBS

M: male; F: female; BMI: body mass index; TC: total cholesterol; TG: triglyceride; LDL: low-density lipoprotein; HDL: high-density lipoprotein; FBS: fasting blood sugar; ↓: decrease; ↑: increase; ↔: no change; TNF-α: tumour necrosis factor-α; PSO: pomegranate seed oil; PSP: pomegranate seed powder; HMWPG: high-molecular-weight polyethylene glycol.

### 5.5. Antioxidant Effects

In a study by Saha and Ghosh [28], streptozotocin-induced diabetic rats treated with α-ESA or PA containing 0.5% total lipids exhibited reduced oxidative stress and increased serum levels of antioxidant enzymes, including superoxide dismutase (SOD), glutathione peroxidase (GPx), and catalase, compared to the control group. This finding was corroborated by their subsequent research on sodium arsenite-induced oxidative stress in rat models [101]. Mukherjee et al. [36] observed a reduction in the peroxidation of PUFAs in lipids and the formation of free radicals; this effect was attributed to the ability of conjugated double bonds in PA in PSO to neutralise free radicals. Additionally, a diet containing 0.25% CLnAs significantly decreased membrane lipid peroxidation compared to one lacking CLnAs. Further, PSO demonstrated a protective effect on brain activity against oxidative stress in a rat model, evidenced by a reduction in protein carbonylation levels and malondialdehyde concentrations through the prevention of lipid accumulation in the brain, along with an increase in the levels of SOD and GPx [102]. The antioxidant properties of PSO were further attributed to its tocopherol and polyphenolic compound content [103]. While in vitro and animal studies consistently report the antioxidant effects of PA, these findings may not directly translate to clinical benefits in humans. The dosages employed in experimental models are often much higher than those achievable through diet, and the bioavailability remains poorly defined. This highlights the need for mechanistic human studies to validate these effects under physiologically relevant conditions.

While omega-3 PUFAs, such as eicosapentaenoic acid (EPA) and docosahexaenoic acid (DHA), are well-established for their cardioprotective, anti-inflammatory, and antioxidant properties [104,105], PA exhibits comparable bioactivities through distinct mechanisms. Both compounds have been shown to reduce plasma lipids and improve insulin sensitivity; however, PA appears to exert stronger effects on lipid metabolism by activating PPARγ [79] and suppressing lipogenic gene expression [87]. In terms of anti-inflammatory action, omega-3 PUFAs modulate eicosanoid pathways and promote the synthesis of pro-resolving mediators, such as resolvins and protectins [106], while PA reduces inflammation by downregulating pro-inflammatory cytokines (e.g., TNF-α, IL-6) and inhibiting NF-κB activation [92,93]. Furthermore, PA’s triene structure confers high antioxidant capacity by scavenging ROS and enhancing endogenous antioxidant enzyme activity [28], showing similar antioxidant effects to omega-3 PUFAs in vitro and in animals [107,108]. Overall, the distinct yet overlapping mechanisms of action suggest that PA may offer complementary therapeutic potential to omega-3 PUFAs, particularly in the context of metabolic, cardiovascular, and inflammatory disorders.

## 6. Clinical Relevance and Limitations of PA

PA shows considerable promise as a bioactive compound due to its anti-inflammatory, lipid-lowering, antioxidant, and antidiabetic effects demonstrated in preclinical models [9,11,17]. These properties suggest potential applications in the management or prevention of chronic conditions, such as metabolic syndrome, obesity, and cardiovascular disease. However, its clinical application remains limited. To date, human studies are scarce and report inconsistent findings [49,59,71,72,86,87,88,100], with variability in the outcomes likely due to differences in the study design, intervention dose, duration, and population characteristics. Furthermore, the bioavailability and metabolic fate of PA [49], particularly its partial conversion to CLA, complicate the interpretation of its specific effects. Another key limitation lies in the lack of standardised dosing and formulations; optimal intake levels for health benefits are not yet clearly defined, and commercial PA supplements vary widely in concentration and purity. Finally, there is limited understanding of the specific populations who may benefit most from PA supplementation. These challenges underscore the need for rigorously designed clinical trials, improved delivery systems, and clearer regulatory guidance to support its safe and effective use in public health or clinical nutrition.

## 7. Conclusions

The rich bioactive profile of PSO contributes to its high nutritional value and potential health benefits, making it a promising candidate for use as an active ingredient. Emerging research provides substantial evidence that PA (a CLnA) may serve as a bioactive fatty acid with significant benefits for cardiometabolic health. It may also offer protective effects against various diseases, including CVD and diabetes, and even enhance their treatment. Although it offers similar advantages to omega-3 PUFAs, PA may provide distinct or even synergistic advantages due to its alternative mechanisms of action. Based on the current evidence, PA needs more emphasis as a complementary agent in preventive nutrition and chronic disease management. However, without robust human clinical trials, its integration into public health strategies remains speculative. Future research should prioritise elucidating its metabolic fate, optimal dosage, and long-term safety in human populations to unlock its full clinical potential.

## Figures and Tables

**Table 1:** Common conjugated linolenic acid (CLnA) fatty acids from plants and their isomeric structures.

Common Name	Isomeric Formula and Chemical Structure	Amount (%)	Reference
Pomegranate seed (*Punica granatum* L., Lythraceae) and Trichosanthes seed (*Trichosanthes kirilowii* Maxim., Cucurbitaceae)	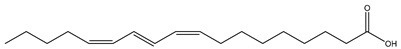 (Punicic acid) C_18:3 c9,t11,c13_	>70% >40%	[45]
Catalpa seed (*Catalpa ovata* G. Don, Bignoniaceae)	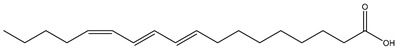 (Catalpic acid) C_18:3 t9,t11,c13_	>40%	[40]
Tung tree seed (*Aleurites fordii* Hemsl., Euphorbiaceae)	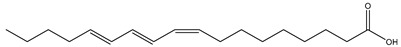 (α-Eleostearic acid) C_18:3 c9,t11,t13_	>70%	[45]
Marigold seed (*Calendula officinalis* L., Asteraceae)	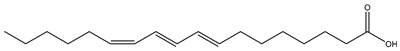 (Calendic acid) C_18:3 t8,t10,c12_	>50%	[39]
Jacaranda seed (*Jacaranda mimosifolia* D. Don, Bignoniaceae)	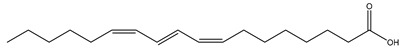 (Jacaric acid) C_18:3 c8,t10,c12_	36%	[38]

## Abbreviations

ApoB100	Apolipoprotein B100
BMI	Body Mass Index
Caco-2	Human Epithelial Colorectal Adenocarcinoma Cells (commonly used as a model of the intestinal barrier)
CD	Conjugated Diene
CFA	Conjugated Fatty Acid
CLA	Conjugated Linoleic Acid
CLnA	Conjugated Linolenic Acid
CVD	Cardiovascular Disease
GPx	Glutathione Peroxidase
HDL-C	High-Density Lipoprotein Cholesterol
ICR	Institute of Cancer Research (mouse strain)
IL	Interleukin
LA	Linoleic Acid
LDL-C	Low-Density Lipoprotein Cholesterol
NF-κB	Nuclear Factor Kappa B
PA	Punicic Acid
PPAR-γ	Peroxisome Proliferator-Activated Receptor Gamma
PS	Pomegranate Seed
PSO	Pomegranate Seed Oil
PSP	Pomegranate Seed Powder
PUFA	Polyunsaturated Fatty Acid
SOD	Superoxide Dismutase
TAG	Triacylglycerol
TC	Total Cholesterol
TFAs	Trans-Fatty Acids
TG	Triglycerides
TNF-α	Tumour Necrosis Factor Alpha
α-ESA	Alpha-Eleostearic Acid
α-LnA	Alpha-Linolenic Acid
